# Hyper-IgD syndrome: a new mutation (p.R277G) with a severe phenotype

**DOI:** 10.1186/1546-0096-9-S1-P21

**Published:** 2011-09-14

**Authors:** J Santos, JI Arostégui, MJ Brito, C Neves, M Conde

**Affiliations:** 1 Pediatric Department, Hospital Dona Estefânia – CHLC – EPE, Lisbon, Portugal; 2 Unidad de Enfermedades Autoinflamatorias, Hospital Clínic, Barcelona, Spain; 3 Pediatric Infectious Diseases Unit, Hospital Dona Estefânia – CHLC – EPE, Lisbon, Portugal; 4 Pediatric Rheumatology, Hospital Dona Estefânia – CHLC – EPE, Lisbon, Portugal

## Background

Hyperimmunoglobulin D Syndrome (HIDS is a rare autosomal recessive condition caused by mutations in the Mevalonate Kinase (MVK) gene that codes for MVK, an essential enzyme in the isoprenoid pathway. It shows a wide phenotypic spectrum probably related with the residual activity of this enzyme (1–20% of normal). So far, about 63 mutations have been described and the four most prevalent mutations account for more than 70%.

## Aim

Case report of a new mutation of the MVK gene in a child with a severe Hyper-IgD syndrome.

## Results

A 2-year-old portuguese boy presented with recurrent episodes of fever and malaise, cervical lymphadenopathy and hepatosplenomegaly since 12 months of age. Rash was seen once. During acute attacks laboratory evaluation demonstrated microcytic hypochromic anemia (Hg 8-10 g/dl), leukocytosis with neutrophilia (WBC 23 490-32 280/mm^3^) and increased ESR (61-91 mm/h), CRP (6.27-18.49 mg/dl) and serum amyloid A (104-510 mg/L). Clinical and laboratory improvement was seen between attacks. Renal and liver functions, like cardiac and ophthalmologic evaluations, were normal. Infectious causes, primary immunodeficiency, neoplastic and autoimmune diseases were excluded. Despite normal serum IgD, HIDS was clinically suspected. DNA sequence analysis revealed a homozygous Arg-277-Gly (p.R277G) new mutation in MVK gene. The healthy non-consanguineous parents were heterozygous for this mutation. Short NSAIDs and corticosteroid courses were given during attacks with poor benefits. Anakinra was started (2mg/Kg/day), with partial clinical response.

## Discussion

R277G is a previously unreported MVK mutation and in this case was associated with a severe phenotype. Further studies are needed to evaluate a co-relation genotype-enzyme activity-phenotype and by so helping to define best therapeutic strategies for each patient.

